# Chemical and microstructural evaluation of dental ceramics before and after multiple firing cycles

**DOI:** 10.1590/0103-644020256637

**Published:** 2025-12-08

**Authors:** Leandro Medeiros Santos, Alberto Nogueira da Gama Antunes, Pedro Giorgetti Montagner, Antônio Marcos Montagner, Rafael Leonardo Xediek Consani, João Maurício Lima Figueiredo Mota

**Affiliations:** 1 Department of Restorative Dentistry, Dentistry School, Federal University of Minas Gerais, Belo Horizonte, MG, Brazil; 2 Department of Dentistry, Pontifical Catholic University of Minas Gerais, Belo Horizonte, MG, Brazil; 3 Department of Prosthodontics and Periodontology, Piracicaba Dental School, State University of Campinas, SP, Brazil

**Keywords:** Dental ceramics, scanning electron microscopy, crystallinity

## Abstract

The study evaluated the leucite content in the powder of EX-3, Cerabien, and Omega 900 dental ceramics, as well as in samples subjected to 3, 8, and 13 firing cycles. Chemical composition of the ceramics before the sintering process and after firing cycles was verified. SEM analyses, combined with energy-dispersive X-ray microspectroscopy and X-ray diffraction, were conducted on unfired powder and sintered samples to determine the semi-quantitative chemical composition. Origin 5.0 software and Peak fitting 5.0 were used to quantify the crystalline phase. EX-3, Cerabien, and Omega 900 ceramics showed the following chemical elements in decreasing average order (%): O, Si, K, Al, Na, Ca, and Mg. Omega 900 showed Ba as an intermediary between Na and Ca. Ceramics also showed the oxides: SiO2, K2O, Al2O3, Na2O, CaO, MgO, and BaO. EX-3 showed leucite and cristobalite crystalline phases, Cerabien cristobalite crystalline phase, and Omega 900 leucite. EX-3 showed a significant increase in leucite content between 3 and 8, as well as between 8 and 13 firing cycles (p<0.05). Cerabien showed a significant increase in cristobalite content between 3 and 8 firing cycles, while Omega 900 showed a significant increase in the leucite content between 3 and 8 firing cycles. Significant decrease between 8 and 13 firing cycles was shown (p<0.05). In conclusion, firing cycles did not change the ceramics chemical compositions, showing microstructure with amorphous and crystalline phases. Ex-3 showed leucite and cristobalite phases, Cerabien cristobalite phase, and Omega 900 leucite phase. The increase in firing cycles promoted changes in the ceramic crystalline phase.

## Resumo

O estudo avaliou o teor de leucita no pó das cerâmicas odontológicas EX-3, Cerabien e Omega 900, bem como em amostras submetidas a 3, 8 e 13 ciclos de queima. A composição química das cerâmicas antes do processo de sinterização e após os ciclos de queima foi verificada. Análises MEV combinadas com micro espectroscopia de raios X por dispersão de energia e difração de raios X foram conduzidas em pó não queimado e amostras sinterizadas para determinar a composição química semi-quantitativa. O software Origin 5.0 e o Peak Fitting 5.0 foram usados para quantificar a fase cristalina. As cerâmicas EX-3, Cerabien e Omega 900 apresentaram os seguintes elementos químicos em ordem média decrescente (%): O, Si, K, Al, Na, Ca e Mg. Omega 900 apresentou Ba intermediário entre Na e Ca. As cerâmicas também apresentaram os óxidos: SiO2, K2O, Al2O3, Na2O, CaO, MgO e BaO. EX-3 apresentou fases cristalinas de leucita e cristobalita, fase cristalina de cristobalita Cerabien e leucita Omega 900. EX-3 apresentou aumento significativo no teor de leucita entre 3 e 8, bem como entre 8 e 13 ciclos de queima (p<0,05). Cerabien apresentou aumento significativo no teor de cristobalita entre 3 e 8 ciclos de queima, enquanto Omega 900 apresentou aumento significativo no teor de leucita entre 3 e 8 ciclos de queima. Foi apresentada diminuição significativa entre 8 e 13 ciclos de queima (p<0,05). Em conclusão, os ciclos de queima não alteraram as composições químicas da cerâmica, apresentando microestrutura com fases amorfas e cristalinas. Ex-3 apresentou fases de leucita e cristobalita, fase de cristobalita Cerabien e fase de leucita Omega 900. O aumento dos ciclos de queima promoveu alterações na fase cristalina da cerâmica.

**Figure:**
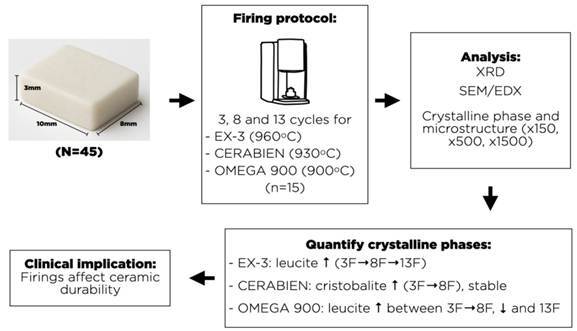

## Introduction

Leucite is a potassium-aluminum-silicate mineral (K₂O·Al₂O₃·4SiO₂) with a primary crystalline phase in dental ceramics for porcelain-fused-to-metal restorations. Zircônia has been introduced in prosthetic dentistry for the fabrication of crowns and fixed partial dentures in combination with CAD/CAM techniques. The processing techniques are assessed in the light of their possible clinical implications and consequences on the long-term performance of zircônia ^1^, whereas repeated heat-pressing treatment produces a significant increase in the flexural strength of glass-ceramic material ^2^. However, other practical aspects are necessary regarding the choice and use of dental ceramics to maximize aesthetics and durability ^3^, since the flexural strength slightly increases with increasing the soak time and temperature ^4^.

Zircônia was introduced in the composition of other ceramics to increase the thermal expansion coefficient of feldspathic glasses (7 × 10⁻⁶ K⁻¹) up to a range similar to that of metal alloys (13 to 15 × 10⁻⁶ K⁻¹). This material is the strongest of the dental ceramics and is being fabricated in monolithic form for a range of clinical applications, and Y-TZP (yttria-stabilized tetragonal zircônia polycrystal) is the most widely used variant. However, current Y-TZP ceramics do not show the aesthetics of competitive glass-ceramics and are therefore somewhat restricted in the anterior region. This process prevents the separation of the ceramic/metal interface during the cooling process, since leucite exhibits values on the order of 22 × 10⁻⁶ K⁻¹ ^5^, a situation that positively impacts the clinical durability of metal-ceramic restoration in the long term. CAD/CAM ceramics are emerging as the material of choice for many restorations and dental appliances. However, it is important to ensure that proper clinical research based on evidence confirming the success and durability of these materials is available before patient care ^6^.

It is a remarkable variation among ceramic compositions of the leucite content in unheated ceramic powders (14 to 32 wt.%) and in the respective sintered powders (15 to 41 wt.%). The low fusing glass-ceramic and the high fusing leucite-based ceramic show significantly higher fracture toughness and microhardness and lower modulus of elasticity compared with the low fusing feldspathic ceramic ^7^. On the other hand, different surface treatments of CAD-CAM monolithic zirconia-reinforced lithium silicate and lithium disilicate glass-ceramics resulted in clinically acceptable color changes after coffee thermocycling. Lithium disilicate was more translucent than zirconia-reinforced lithium silicate before and after coffee thermocycling ^8^.

SEM investigation revealed that the different firing parameters had an impact on the alloy, the ceramic microstructure, and the surface quality. In addition, the differences in the chemical composition of the ceramics, as shown by EDS, are reflected in their behavior, since the crystalline alloy structure is influenced by the repeated firings of the ceramic layers. Therefore, it must be considered the sensible statement that the crystalline phases in dental ceramics must be kept stable during material processing and during use in the oral environment ^9^.

Dental ceramics are frequently subjected to a high number of firings during processing to obtain satisfactory aesthetics in indirect dental restorations. Ceramic fired at 900°C showed superior mechanical and abrasion resistances, and SEM analysis depicted a homogeneous mass of dental porcelain, implying that the material firing was complete at this temperature ^10^. However, the amount of leucite in the composition of the ceramics can be affected by multiple firing cycles ^11^, sintering of SiO2Al2O3-K2O glass powder at 700 to 850°C/1 to 12 h contributed to the crystallization of cubic leucite and sanidine with minor phases of microcline ^12^, and significantly improves the marginal fit of conventionally sintered and speed-sintered monolithic zircônia crowns ^13^.

An interesting fact was that the results of the four-point flexure test were significantly lower than those obtained in the three-point flexure test for veneering ceramics for the metal-ceramic technique, while the four-point flexure test showed the highest discrimination between the different ceramic materials ^14^. Despite this, clinical data suggest that zirconia-based fixed dental prostheses may serve as an alternative to metal fixed dental prostheses ^15^.

Based on these considerations, the aim of this study was to verify: 1- The semi-quantitative chemical composition through scanning electron microscopy analysis associated with energy-dispersive X-ray spectroscopy before and after multiple firing cycles. 2- The amorphous and crystalline phases by X-ray diffraction of the EX-3, Cerabien, and Omega 900 ceramics before processing and after being subjected to 3, 8, and 13 firing cycles. The study hypothesis was that the multiple firing cycles would significantly affect the chemical composition, microstructure, and quantity of crystalline phases (leucite and cristobalite).

## Materials and methods

The EX-3 (Noritake, Nagoya, Japan), Cerabien (Noritake, Nagoya, Japan), and Omega 900 (Vita Zahnfabrik, Bad Säckingen, Germany) ceramic samples with 10 x 8 x 3 mm dimensions (n=45) were prepared in refractory investment molds (Nori-Vest; Noritake) filled with the ceramic slurry (powder/distilled water), condensed in bench vibrator operating at a 60 Hz frequency (Master Plaster Vibrator; Biotron, Santa Rita do Sapucaí, MG, Brazil) with low-medium amplitude for ~30-60 s until a homogeneous slurry was obtained. Excess slurry surface moisture was removed with absorbent paper before firing. The amorphous and crystalline phases of the ceramics were analyzed by X-ray diffraction.

Refractory molds filled with the ceramic were fired according to the manufacturer’s instructions: EX-3 - 960 °C, Cerabien - 930 °C, and Omega 900 - 900 °C in a furnace (Vista, Jelrus International Inc., Hicksville, NY, USA) according to the parameters recommended by the ceramic manufacturers (Table 1). After the firing cycles, the samples were abraded with discs (30 to 40 µm) and polished with 200, 400, 600, and 1000 sandpaper grids (Aropol 2V Polisher; Arotec, Sao Paulo, SP, Brazil). The samples were cleaned with a steam water device (Vapor-Jet II, EDG, Equipment and Controls, Sao Carlos, SP) to remove residues from the polishing process. After, the samples were washed with running water, rinsed in deionized water for three periods of five minutes, ultrasonically cleaned with 70% isopropyl alcohol (Ultrasonic Cristofoli Biosafety; Campo Mourao, PR, Brazil), and dried and stored at room temperature for 48 h. The samples from each ceramic (n=15) were submitted to 3, 8, or 13 firing cycles (n=5).

**Table 1:** Firing cycle parameters recommended by manufacturers.

Ceramic	Step	Pre-dry (°C/min)	Start vacuum (°C)	Heat rate (°C/min)	Vacuum (level/time)	Release vacuum (°C)	Peak (°C)	Hold at Peak (min)
Super Porcelain EX-3 (Kuraray Noritake)	Body / Enamel	600 / 7	600	45	~96 kPa	~920	~930	0
	Glaze / External stain + glaze	650 / 5	-	50	no vacuum	-	~910	0
Cerabien (Kuraray Noritake)	Body / Enamel	600 / 7-10	600	45	81-96 kPa	~930-940	~930-940	~1 (in air)
	Glaze (self-glaze / FC Paste Stain glaze)	600 / 5	-	45-50	no vacuum	-	~910	~0-1
Vita Omega 900 (VITA Zahnfabrik)	1st dentine firing	600 / 6	-	50	vacuum ~6 min	-	900	1
	2nd dentine firing	600 / 6	-	48	vacuum ~6 min	-	890	1
	Glaze	600 / 0-4	-	75	no vacuum	-	900	1-2

The X-ray diffraction analysis was made with a diffractometer (PW1710; Malvern Panalytical, Phillips, Netherlands) with copper Kα radiation and a curved graphite monochromator. Data were collected from 15 to 40° at 0.05 2θ/s. The crystalline phases identification was performed comparing the diffraction peaks mainly for leucite (KAlSi₂O₆) and cristobalite (SiO₂) displayed on the current analyses with the results from the ICDD 2001 - International Center for Diffraction Data ^16^. The crystalline phases identified in the current analyses were determined using the integrated intensity measurement through Origin 5.0 (Microsoft, USA) and Peak fitting 5.0 (Microsoft, USA) software. ANOVA two-way was taken to verify the association between the area below the high-intensity Peak from the primary crystalline phase and the number of firing cycles. When a significant effect was revealed, Duncan’s multiple range test was applied to compare the number of firing cycles (3, 8, or 13).

Samples microstructural morphological analysis was performed with scanning electron microscopy (JEOL 5410, Peabody, Massachusetts, USA) associated with energy-dispersive X-ray micro spectroscopy. The samples were evaluated after firing to observe the surface of the materials and identify the ceramic chemical composition. The samples submitted to 8 firing cycles were placed on stubs and coated with gold in a vacuum cathodic spray device and analyzed in a secondary electron detector (Carl Zeiss, Oberkochen, Germany) at 15.0 kV, with a reading distance of 8 mm and 1600x magnification.

## Results

Table 2 shows the chemical composition of the powders of the EX-3, Cerabien, and Omega 900 ceramics. Table 3 shows the chemical composition of the ceramics after eight firing cycles. Table 4 shows the ANOVA 2-way repeated measures for ceramics after multiple firings.

**Table 2:** Oxide chemical composition of the ceramic powder (weight %).

Ceramic\Oxide	SiO_2_	K_2_O	Al_2_O_3_	Na_2_O	Cao	MgO	BaO
Ex-3	58.80	17.19	12.45	9.56	1.26	0.71	0.0
Cerabien	73.14	11.,47	8.04	5.95	0.96	0.43	0.0
Omega 900	50.15	18.31	13.04	8.11	2.54	0.50	7.34

**Table 3:** Oxide chemical composition of the ceramic after 8 firing cycles (weight %).

Ceramics/Oxide	SiO_2_	K_2_O	Al_2_O_3_	Na_2_O	CaO	MgO	BaO
Ex-3	59.31	19.82	12.20	7.21	1.10	0.36	0.0
Cerabien	74.00	11.92	7.81	5.14	0.69	0.44	0.0
Omega 900	53.77	17.52	13.39	5.76	2.64	0.42	6.50

**Table 4:** ANOVA 2-way repeated measures.

Source	Sum of squares	df	Mean Square	F	P value
Ex-3	59.31	19.82	12.20	7.21	1.10
Cerabien	74.00	11.92	7.81	5.14	0.69
Omega 900	53.77	17.52	13.39	5.76	2.64

**Figure 1:**
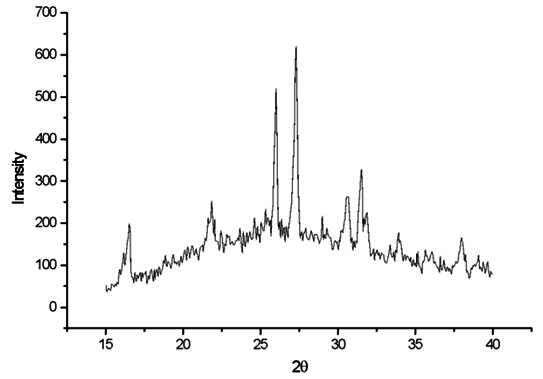
X-ray diffraction pattern for EX-3 ceramic.

Figure 1 shows the X-ray diffraction pattern for EX-3 ceramic. Amorphous phase and crystalline peaks related to the tetragonal leucite and cristobalite are shown. The peaks with 2θ values at 27.27° and 25,89° are the leading peak positions of tetragonal leucite. The peaks observed at 2θ values of 21.76° and 35.91° are the leading peak positions of cristobalite.

**Figure 2:**
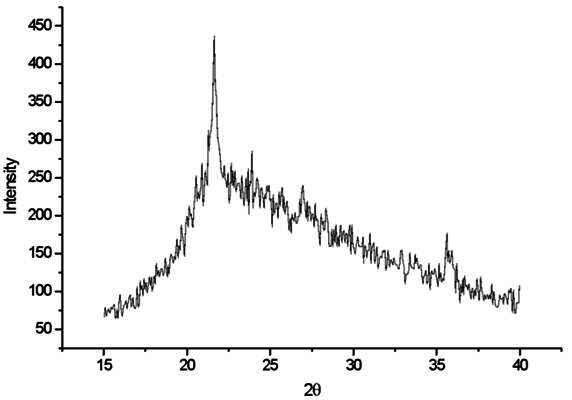
X-ray diffraction pattern diagram for Cerabien ceramic.

Figure 2 shows the Cerabien and Omega 900 ceramic crystalline phases. Amorphous phase and cristobalite crystalline phase for Cerabien are identified with peaks at 2θ values of 21.76° and 35.91° as the leading cristobalite peak positions.

**Figure 3:**
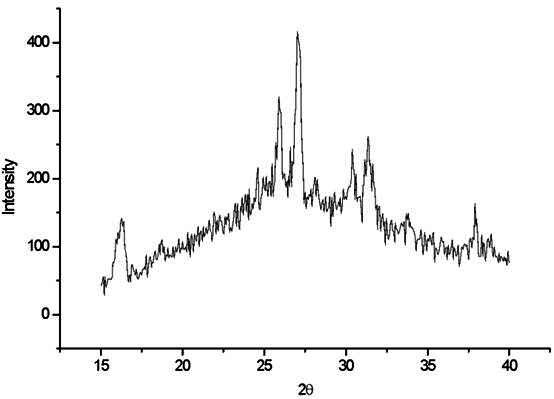
X-ray diffraction pattern for Omega 900 ceramic.

Figure 3 shows the Omega 900 ceramic crystalline phases. The amorphous phase and leucite crystalline phase identify the crystalline tetragonal leucite with peaks at 2θ values of 27.27° and 25.89°, respectively, as the leading tetragonal leucite peak positions.

**Figure 4:**
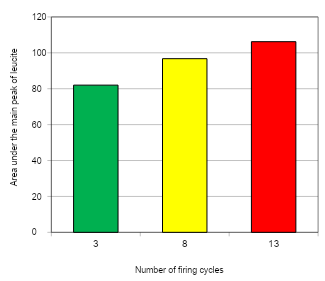
Number of firing cycles/average area of the central peak of leucite for EX-3 ceramic.

**Figure 5:**
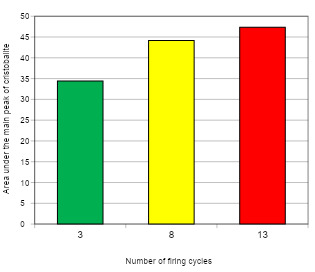
Number of firing cycles/average area of the central peak of leucite for Cerabien ceramic.

**Figure 6:**
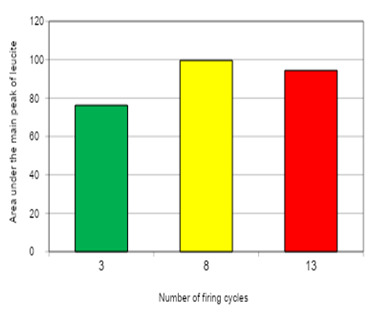
Number of firing cycles/average area of the central Peak of leucite for Omega 900 ceramic.

Data from quantitative analysis of the crystalline phases of the ceramics submitted to the increase of the number of firing cycles (XDR) are shown in Figures 4, 5, and 6. Analysis showed that the crystalline phases observed in the unfired powder were also identified after multiple firings, and no other crystalline phase was detected. A significant effect between the mean area under the central peak of leucite and the number of firing cycles was revealed by ANOVA (p<0.05). Duncan's multiple range test showed that the increase in the number of firing cycles from 3 to 8 promoted an increase of 17.97% in the area under the central leucite peak. The increase from 8 to 13 firing cycles promoted an increase of 9.79% and the increase from 3 to 13 promoted an increase of 9.52% in the area under the central leucite peak (Figure 4).

A significant effect between the area under the central leucite peak and the number of firing cycles was shown by ANOVA (p<0.05). Duncan’s multiple range test showed that the increase in the number of firing cycles from 3 to 8 promoted an increase of 30.69% in the area under the central leucite peak. The increase from 8 to 13 firing cycles promoted a decrease of 5.27% and the increase in the number of firing cycles from 3 to 13 promoted an increase of 23.80% in the area under the central leucite peak (Figure 6).

**Figure 7:**
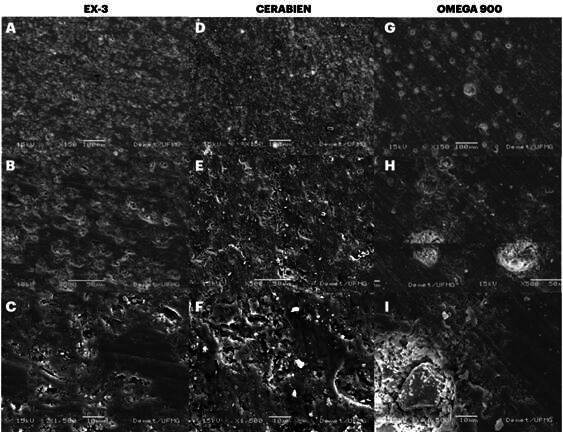
MEV illustrations: EX-3 (A, B, C), Cerabien (D, E, F), and Omega 900 (G, H, I). (A, D, G - 150x), (B, E, H - 500x), and (C, F, I - 1500x), identifying the amorphous and crystalline phases at 8 firing cycles.

Figure 7 shows the MEV illustrations identifying the amorphous and crystalline phases after 8 firing cycles: EX-3 (A, B, C), Cerabien (D, E, F), and Omega 900 (G, H, I) with 150, 500, and 1500x magnifications, respectively. The firing process step in which the ceramic temperature is reduced to prevent cracking, warping, or other failures due to thermal stresses is known as cooling or tempering. The typical clinical occurrence of multiple refutations of ceramic indirect restoration due to excessive firing cycles was arbitrarily represented in this study by the 8-fire cycle (value between 5 and 13 firing cycles).

SEM analysis reveals differences in the microstructure among the three ceramics, specifically in the distribution of amorphous and crystalline phases. EX-3 (Figure 7A-C) shows a homogeneous and smoother surface, probably due to a predominantly glassy matrix with minimal crystalline regions. Cerabien (Figure 7D-F) displays a rougher and heterogeneous microstructure with clusters of crystalline phases, probably leucite integrated with the glassy matrix. Omega 900 (Figure 7G-I) shows a crystalline structure with large aggregates that suggest a higher level of crystallinity.

## Discussion

This study evaluated the leucite content in the unfired powder of EX-3, Cerabien, and Omega 900 dental ceramics, and in the samples subjected to 3, 8, and 13 firing cycles. The chemical composition of the ceramics before the sintering process and after 8 firing cycles was also verified. The study hypothesis was that the multiple firing cycles would significantly affect the chemical composition, microstructure, and quantity of ceramic crystalline phases (leucite and cristobalite).

Table 3 shows the chemical composition of the fired ceramics using EDX analyses. It is possible to verify that silicon is the oxide with the highest percentage in the evaluated ceramics. This result agrees with a previous study showing a significant amount of SiO₂ in dental ceramics ^1^. However, silicon oxide in Cerabien showed a higher proportion (74%) compared with EX-3 (59.31%) and Omega 900 (53.77%). This fact probably occurred due to the nature of the different crystalline phases, which was also verified by the XRD analysis in previous studies ^6^ ^,^ ^10^.

In addition, it is evident that in these ceramic material types, both the crystalline and the amorphous phases are composed of SiO.K₂O, components that correspond to the second most observed oxide in the ceramics analyzed in the current study, with average values of 19.82% for EX-3, 11.92% for Cerabien, and 17.52% for Omega 900. As alleged in a previous study ^4^, the proportion of leucite in the ceramic after processing is determined by the amount of K₂O, and is a crucial factor in achieving thermal compatibility between ceramic/metal alloy. The highlighting of the importance of K₂O content in determining the thermal properties of dental ceramics was also shown in a previous study ^8^.

Most of the dental ceramics use leucite to adjust the coefficient of thermal expansion of the material. For this reason, the composition of the crystalline phases of ceramics has been evaluated in previous studies, considering different variables ^3^ ^,^ ^7^ and emphasizing the effect of the leucite in enhancing the mechanical strength due to the reinforcement provided by the crystals dispersed within the glassy ^7^. In the current study, the cristobalite was the only crystalline phase observed in the Cerabien ceramic, without any traces of leucite or any other crystalline phase. Ceramics with high SiO₂ concentration, with cristobalite predominating after the firing cycles ^9^, showed similar results compared with the current study. In addition, a previous study alleged that the absence of leucite can affect the thermal and mechanical properties of dental ceramics ^15^. According to ICDD 88-2302 ^16^, the prominent peaks of the quartz phase are found at 2θ = 26.64° and 2θ = 20.86°, corresponding to d(Å) = 3.34 and d(Å) = 4.25, respectively.

The peaks shown in the XRD analysis of the EX-3 and Cerabien ceramics belonged to the cristobalite phase according to ICDD 82-0512 ^17^. The most intense peaks are shown at 2θ = 21.76° and 35.91°, corresponding to d(Å) = 4.08 and 2.49, respectively, as also observed in the current study (Figure 2). An earlier study showed that the Cerabien ceramic did not show leucite in the composition; therefore, it would not be possible to classify this material as a dental feldspathic ceramic or even a leucite ceramic ^6^. The absence of the leucite phase can also suggest that the thermal expansion coefficient of the ceramic would be lower compared with EX-3 and Omega 900 ceramics. In addition, the ceramics without leucite can have different clinical applications due to the distinct thermal properties ^18^.

The prominent peaks related to tetragonal leucite are observed at 2θ values of 27.27° and 25.89°, corresponding to d(Å) = 3.26 and d(Å) = 3.43, respectively. In the current study, the results obtained from XRD in EX-3 and Omega 900 showed the prominent peaks identified as those of tetragonal leucite, both in the unfired powder and in the samples subjected to multiple firing cycles. The prominent peaks related to cubic leucite were not observed, agreeing with previous findings ^4^ ^,^ ^19^ ^,^ ^20^. On the other hand, it has been alleged that the cubic leucite was identified in feldspathic ceramic-based glass ceramics ^4^ and heat-pressed feldspathic ceramic ^21^, both subjected to different firing protocols. Some authors suggest that the multiple firing cycles can also induce the leucite crystallization in dental ceramics. In addition, a significant increase in the leucite content was shown after multiple firing procedures at the manufacturer's recommended temperature ^4^ ^,^ ^22^.

Data showed that the Cerabien ceramic tends to stabilize the cristobalite level with the increase of the number of firing cycles (Figure 2). Lower rate of crystallization was found when a statistically significant increase in cristobalite content was shown between 3 and 8 firing cycles. However, this result was not observed between 8 and 13 cycles. A previous study also observed similar stabilization in lithium disilicate ceramics after multiple thermal cycles ^9^. The stabilizing cristobalite crystallization in Cerabien ceramic seems similar to the behavior of lithium disilicate after successive firing cycles ^6^. In addition, it was alleged that the microstructural stabilization would be crucial for the predictability of the clinical use of ceramics after multiple thermal processes ^7^. The ceramic microstructural stability may be the reason why different firing protocols did not affect the flexural strength or the surface roughness of the CAD/CAM lithium disilicate glass-ceramic, even after several agings ^23^.

Modifications in thermal processing or changes in the composition of feldspathic glasses can stabilize the cubic leucite in samples fired within the temperature range suggested by the manufacturer, as well as in the ceramic powder before thermal processing ^5^ ^,^ ^24^. The coefficient of thermal expansion of stabilized cubic leucite is significantly lower compared with that of the tetragonal leucite ^1^. Thus, when tetragonal leucite crystals with high thermal contraction are surrounded by the glassy matrix with lower contraction, tangential compressive stress occurs around the crystals, reinforcing the material by deflecting cracks away from the leucite crystals ^2^ ^,^ ^5^ ^,^ ^7^. However, changes in the crystalline form of leucite can create incompatibilities between the ceramic and the metal alloy ^4^.

The XRD analyses in the current study did not show traces of cubic leucite at any stage of the thermal processing. The absence of undesirable phases, such as sanidine, is considered beneficial for maintaining the thermal and mechanical stability of ceramics ^25^. Omega 900 showed complex behavior under multiple firing cycles, with a statistically significant increase in leucite content between 3 and 8 firing cycles. Conversely, a significant decrease in this content between 8 and 13 firings can be observed (Figure 3). This cyclic behavior was also observed in a previous study with leucite ceramics subjected to multiple thermal cycles ^4^.

The trend of crystallization followed by vitrification after multiple firings in pressable glass-ceramics ^2^ and feldspathic-based glass-ceramics ^4^ resulted in behavior similar to that occurring in Omega 900, where the leucite content increased between 1 and 4 firings and decreased after 8 to 16 firings. This chemical behavior might be related to partial solubilization of crystals at high temperatures, followed by recrystallization during cooling ^13^.

Based on the considerations of the articles that support this work, everything indicates that the most important conclusion of the current study would be that the increase in firing cycles promoting changes in the amount of the crystalline phase could impact the level of mechanical strength of the ceramic and consequent clinical implications (roughness, gloss, aesthetic, marginal adaptation, and fractures) in the long term.

However, the study shows limitations that should be acknowledged: The applied model may suggest similarity of results in other dental ceramics and consequent clinical applications. The study only focused on three specific ceramic types, which do not encompass the diversity of materials available in dental practice and the long-term clinical implications. Therefore, further researches are needed to evaluate how these changes may influence the mechanical properties, durability, and performance of dental indirect restorations. In addition, the oral environment is complex and influenced by various factors, which can affect the mechanical ceramic properties.

## Conclusion

Despite the limitations of the study, the following conclusions can be drawn: 1. The firing cycles did not change the EX-3, Cerabien, and Omega 900 ceramics chemical composition. 2. The ceramics showed a microstructure composed of amorphous phase and crystalline phases. 3. Ex-3 showed leucite and cristobalite crystalline phases, Cerabien cristobalite crystalline phase, and Omega 900 leucite crystalline phase. 4. The increase in firing cycles promoted changes in the amount of the ceramic crystalline phase.
